# Metric-based analysis of FTIR data to discriminate tissue types in oral cancer†

**DOI:** 10.1039/d3an00258f

**Published:** 2023-04-10

**Authors:** Barnaby G. Ellis, James Ingham, Conor A. Whitley, Safaa Al Jedani, Philip J. Gunning, Peter Gardner, Richard J. Shaw, Steve D. Barrett, Asterios Triantafyllou, Janet M. Risk, Caroline I. Smith, Peter Weightman

**Affiliations:** a Department of Physics, University of Liverpool L69 7ZE UK peterw@liverpool.ac.uk; b Liverpool Head and Neck Centre, Department of Molecular and Clinical Cancer Medicine, University of Liverpool L7 8TX UK; c Manchester Institute of Biotechnology, University of Manchester 131 Princess Street Manchester M1 7DN UK; d Head and Neck Surgery, Liverpool University Foundation NHS Trust, Aintree Hospital Liverpool L9 7AL UK; e Department of Cellular Pathology, Liverpool Clinical Laboratories, University of Liverpool Liverpool L7 8YE UK

## Abstract

A machine learning algorithm (MLA) has predicted the prognosis of oral potentially malignant lesions and discriminated between lymph node tissue and metastatic oral squamous cell carcinoma (OSCC). The MLA analyses metrics, which are ratios of Fourier transform infrared absorbances, and identifies key wavenumbers that can be associated with molecular biomarkers. The wider efficacy of the MLA is now shown in the more complex primary OSCC tumour setting, where it is able to identify seven types of tissue. Three epithelial and four non-epithelial tissue types were discriminated from each other with sensitivities between 82% and 96% and specificities between 90% and 99%. The wavenumbers involved in the five best discriminating metrics for each tissue type were tightly grouped, indicating that small changes in the spectral profiles of the different tissue types are important. The number of samples used in this study was small, but the information will provide a basis for further, larger investigations.

## Abstract 2

Comparison of (a) H&E image of oral squamous cell carcinoma with (b) ratio of absorbance at 1252 cm^−1^/1285 cm^−1^ and (c) pseudo-coloured labelled image of seven types of tissue found in oral tissue.

## Graphical abstract

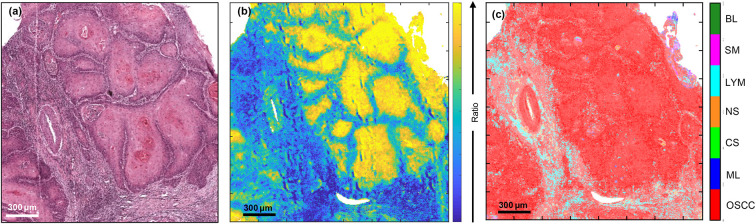

## Introduction

Infrared (IR) techniques have been used to investigate tissue types in a wide range of cancers, particularly Fourier transform infrared (FTIR) spectroscopy and Raman spectroscopy. Although IR spectra are difficult to interpret by direct inspection, significant progress has been made by the application of machine learning techniques.^1–6^ Both FTIR and Raman spectroscopy have been used to study oral squamous cell carcinoma (OSCC) (see Byrne *et al.*^7^ for a review). In particular, Fukuyama *et al.*^8^ reported that the FTIR spectra of normal tissue have stronger contributions from keratin and collagen than abnormal tissue. Bruni *et al.*^9^ noted differences in the FTIR spectra of cancerous and normal oral tissues attributable to collagen, DNA and lipids, whereas Pallua *et al.*^10^ used principal component analysis (PCA) and cluster analysis to produce pseudo-colour images of tissue microarray (TMA) samples and showed correspondence between FTIR and routine histology images.

A machine learning algorithm (MLA) based on analysis of ratios of FTIR absorbance at different wavenumbers, referred to as metrics,^11^ was applied to spectral images of premalignant oral tissue (oral epithelial dysplasia (OED)) and shown to predict malignancy with a sensitivity of 84% ± 3% and a specificity of 79% ± 3%.^12^ This is in agreement with a previous analysis of the same dataset using a PCA-LDA (linear discriminant analysis) approach.^13^ The ability to predict the prognosis of OED is an important advance since while current histopathology techniques can diagnose cancer they cannot predict the prognosis of lesions.^22^ Given the success of the MLA in predicting the prognosis of OED, it is important to investigate its efficacy in more detail. In this work the approach is shown to discriminate between seven classes of tissue related to OED. The analysis provides additional insight into the ability of the MLA to discriminate between lymph node tissue and metastatic oral cancer with sensitivities and specificities of ∼99%.^14^

## Experimental

### Preparation of samples for analysis

Archival blocks of formalin-fixed, paraffin-embedded (FFPE) tissues from five patients with primary OSCC and cervical lymph node metastases, were obtained following informed consent and under ethical approval (REC number EC 47.01).

Regions of interest (ROI) were identified by light microscopy on sections routinely prepared and stained with haematoxylin and eosin (H&E) and included the following types of tissue: OSCC; tumour stroma with immune/inflammatory reaction (CS); non-dysplastic oral epithelium with progenitor (basal/parabasal, BL) and maturation (spinous/keratinised, ML) layers; pre-existing normal stroma (NS); submucosal components (*e.g.* skeletal muscle: SM); and lymphoid node tissue (LYM). Serial, 5 μm sections were cut from the blocks and floated onto charged glass slides for H&E staining and onto calcium fluoride (CaF_2_) disks for FTIR imaging. While sections for H&E were eventually subjected to deparaffinisation, sections for FTIR imaging remained in paraffin wax to minimise further alterations in chemistry and structural organisation of the tissue samples.

For each patient four serial sections were prepared—two sections for FTIR imaging sandwiched between two sections stained with H&E. Images of the H&E stained sections were scanned using an Aperio CS2scanner (Leica Biosystems) to facilitate co-registration and comparison with IR images.

### FTIR experiments

Mid-IR hyperspectral (HS) images were acquired from each ROI using an Agilent Cary 620 FTIR microscope coupled to an Agilent Cary 670 FTIR spectrometer as described previously.^11,15^ In summary, data was collected in transmission from 3800–900 cm^−1^ with a spectral resolution of 4 cm^−1^ and with an effective pixel size of 5.5 μm. Poor quality pixels, defined as having an Amide I absorbance (peak centre 1650 cm^−1^) <0.1 or >2, were removed from the dataset. This range was chosen so that outlier spectra arising from sub-optimal sample thickness would be discarded whilst retaining the vast majority of data. The spectra were then truncated to 900 cm^−1^–1800 cm^−1^ and the region dominated by paraffin (1350 cm^−1^–1500 cm^−1^) was omitted from the analysis. Each spectrum in the truncated dataset was then subjected to a rubber-band baseline correction,^16^ followed by vector normalisation. Corrections for resonant Mie scattering correction were unnecessary for FFPE tissue due to the refractive index matching between the tissue and paraffin.^17^

The H&E and FTIR images were cross-referenced and spectra from the tissue types in each ROI were identified. FTIR datasets were selected from one of each pair of sections cut onto CaF_2_ discs, based on the overall morphological similarity between the FTIR image at 1650 cm^−1^ and the adjacent H&E section, and used to train several multi-class discriminatory models using the MLA.^11^ An equal number of spectra were randomly sampled from each image to mitigate the risk of inducing image-related bias. A five-fold cross validation protocol was employed while training to ensure that all the data available was used to train the model.^18^ To minimise fitting bias, the data was combined from all patients and randomised so that patients were equally represented throughout all stages of the analysis, ensuring that the results of the training and testing stages were as generalised as possible.

### Machine learning algorithm

The metrics-based approach is a supervised machine learning algorithm that characterises biological samples by generating metrics that consist of probability density functions (PDF) that characterise the ratio of absorbance values between two different wavenumbers. Within the training stage a metric is generated for every possible wavenumber combination. The metrics are designed to characterise reliable differences in chemically sensitive IR spectra between different biological samples. These differences ultimately derive from variations in the relative amounts of molecular constituents within the samples. In the testing stage each metric is individually assessed using a scoring mechanism to identify the most effective metrics for discriminating between the samples. This is done by testing each metric's ability to correctly identify spectra not used within the prior training stage. This results in an area under (AUC) receiver operating characteristic (ROC) curve for each metric, indicating an overall classification performance with regards to sensitivity and specificity. The metrics with the highest scores are then assembled into an optimal set tailored for discriminating between the samples which highlights the critical wavenumbers required for accurate classification. The PDFs associated with the metrics in the optimal set, along with a committee-based voting system, can be used as a model capable of predicting the classification of any spectrum. The efficacy of the final model is demonstrated by labelling spectra not used in either of the previous stages to give an overall measure of the model's performance.

### Pseudo-colouring of FTIR images

Spectra contained within FTIR images that were not used in the training were labelled using the predictive model produced by the MLA, assigning both a tissue type and a corresponding confidence value for the prediction, and producing a pseudo-coloured image as follows. The MLA processed each spectrum individually and output the probability of it belonging to each of the tissue types with which the MLA was trained. The tissue type with the highest probability was selected as the spectrum's predicted label. The confidence value was calculated by dividing the highest probability by the sum of all the calculated probabilities. A combined image could thus be formed with each pixel pseudo-coloured according to the tissue label and with a colour saturation determined by the confidence value.

## Results

### Application of the previously described 1252 cm^−1^/1285 cm^−1^ discriminatory metric to primary OSCC and oral epithelium

**Fig. 1:**
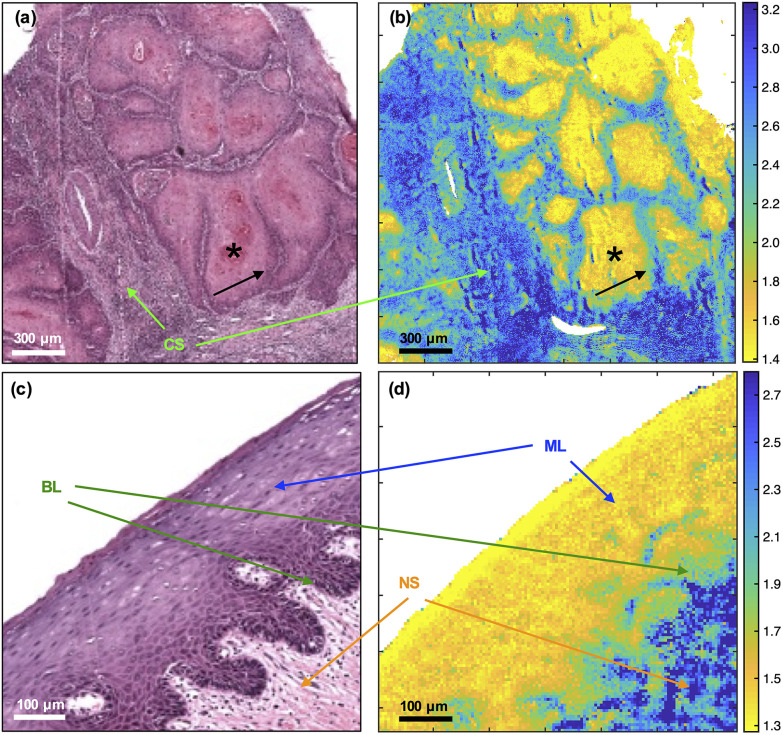
Comparison of H&E (left) with FTIR ratio at 1252 cm^−1^/1285 cm^−1^ (right) images of OSCC (top) and oral epithelium (bottom). See Experimental for explanation of tissue type abbreviations. Keratinising cores are indicated with asterisks and the peripheries of the tumour with black arrows.

In primary OSCC, the centres (cores) of tumour-cell aggregates were identified with high confidence (yellow) in comparison with the inflammatory reaction in the tumour stroma (blue). The metric was less efficient at identifying the periphery (front) of the aggregates (green) [Fig. 1(a) and (b)]. In oral epithelium, the metric also highlighted the maturation layers (yellow) and, to a lesser extent, the progenitor layers (green) [Fig. 1(c) and (d)].

The tumour-cell aggregates in Fig. 1(a) show variably eosinophilic, keratinising cores (asterisk) staining in shades of pink/red and haematoxyphilic, non-keratinising, purple staining periphery (arrow); and separated by tumour stroma with a brisk, heavily haematoxyphilic and purple staining immune/inflammatory reaction. A vessel is shown at the left centre of Fig. 1(a) and (b). Only a sprinkling of inflammatory cells is seen in the pre-existing stroma in Fig. 1(b). Similarities in pseudo-colouring are evident between the core of tumour-cell aggregates and maturation layers of oral epithelium (ML) and between the periphery (front) of tumour-cell aggregates and progenitor layers of oral epithelium (BL).

### Discrimination between tissue types

The 1252 cm^−1^/1285 cm^−1^ metric seemed less discriminatory in the context of multiple tissue types and hence the MLA was also trained to discriminate between tissue types defined in Experimental and to identify other metrics allowing an insight into the molecular composition of those tissues. The algorithm was able to discriminate simultaneously between the various tissue types, though with differing efficacy (Table 1).

**Table 1:** Number of spectra, mean sensitivity and specificity for each tissue type

Tissue	No of spectra (no of images)	Sensitivity (%)	Specificity (%)
Oral squamous cell carcinoma	33534 (3)	82.4 ± 0.6	92.4 ± 0.3
Tumour stroma with immune/inflammatory reaction	1897 (1)	91.5 ± 1.0	99.5 ± 0.6
Progenitor layers of normal epithelium	2036 (2)	93.6 ± 1.3	82.9 ± 0.9
Maturation layers of normal epithelium	5691 (3)	91.1 ± 0.9	95.2 ± 0.3
Pre-existing normal stroma	21752 (4)	95.1 ± 0.4	92.3 ± 0.2
Submucosal components	14790 (2)	83.2 ± 0.7	86.4 ± 1.0
Lymphoid node tissue	4322 (2)	96.2 ± 1.0	94.1 ± 0.8

**Fig. 2:**
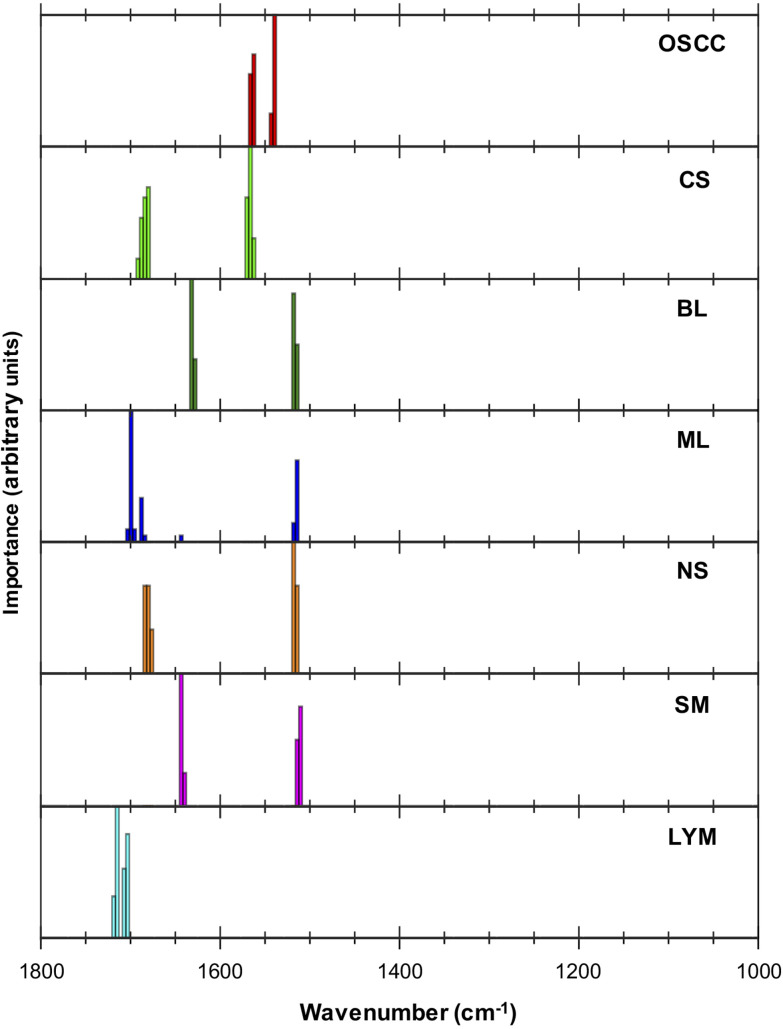
Importance plot of the five highest ranked metrics. Importance is defined in terms of the contribution made to discriminating a particular tissue type from the others. The height of the histogram represents how frequently that wavenumber appears within the five highest ranked discriminatory metrics for the particular tissue.

Examination of the five most discriminating metrics for each tissue type (Table 2) suggested that some wavenumbers were characteristic (Fig. 2). For example, 1539 cm^−1^ and 1562 cm^−1^ characterised OSCC due to the well-separated distributions of ratio values as shown in ESI Fig. 1.† Wavenumbers 1703 cm^−1^ and 1715 cm^−1^ characterised lymphoid node tissue. Other wavenumbers appeared to be shared between different metrics that discriminated between normal and malignant tissues, *e.g.*, wavenumbers 1514 cm^−1^–1510 cm^−1^ in normal tissues (ML, BL, NS, SM).

**Table 2:** The top five ranked metrics discriminating each tissue from the others

OSCC	CS	BL	ML	NS	SM	LYM
1562/1539	1570/1684	1518/1632	1699/1514	1514/1684	1643/1514	1715/1703
1539/1562	1566/1684	1632/1518	1514/1699	1514/1680	1643/1510	1703/1715
1566/1539	1570/1680	1628/1518	1688/1699	1518/1684	1514/1643	1703/1719
1539/1566	1566/1680	1518/1628	1696/1514	1518/1680	1510/1643	1707/1715
1562/1543	1570/1688	1514/1632	1703/1643	1518/1676	1640/1510	1715/1707

**Fig. 3:**
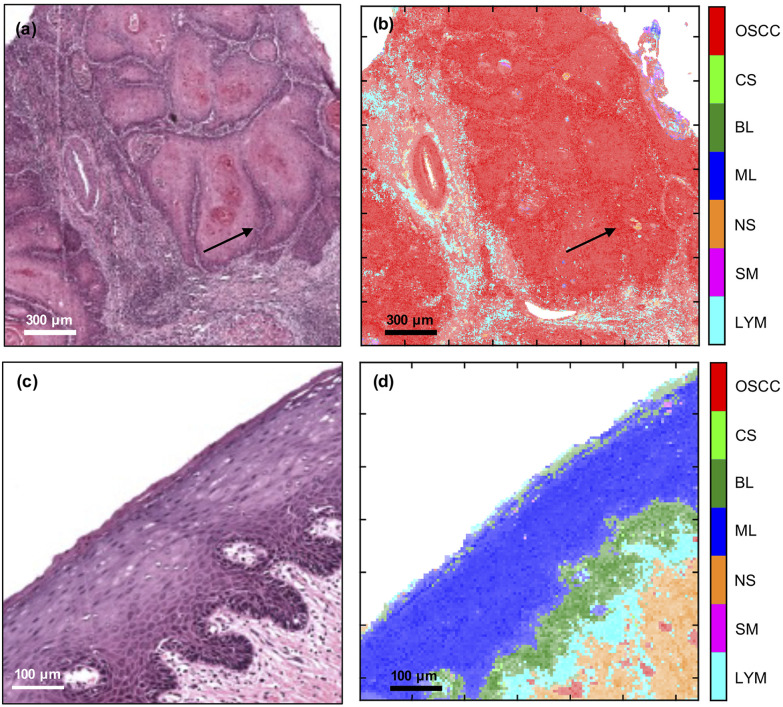
Comparison of H&E (left) with FTIR-HS pseudo-colour (right) images of OSCC (top) and oral epithelium (bottom). The saturation of the pixels in (b) and (d) correspond to the confidence in labelling the tissues by the MLA (more detail in ESI Fig. 2†). The arrows indicate the periphery of the tumour.

The trained MLA model can be used to classify each spectrum in an FTIR-HS image previously unseen by the MLA and construct a pseudo-colour map showing the tissue label and its corresponding confidence value (Fig. 3). The figure indicates that the multi-class analysis using the MLA discriminated between the different tissue types more accurately than the single FTIR ratio at 1252 cm^−1^/1285 cm^−1^. In the primary tumour, OSCC status is assigned; the peripheries of tumour aggregates were more confidently allocated than their more differentiated centres [compare the pseudo-colour intensity at the periphery of the aggregates compared with the cores in Fig. 3(b)]. No component of the oral epithelium is assigned OSCC status [red, Fig. 3(b)] and no component of the tumour is assigned normal epithelial status [blue or green, Fig. 3(d)]. More significantly, there was no obvious cross-over between the progenitor layers of oral epithelium and the periphery (front) of tumour cell aggregates [compare Fig. 3(b) and (d) with Fig. 1(b) and (d)].

**Fig. 4:**
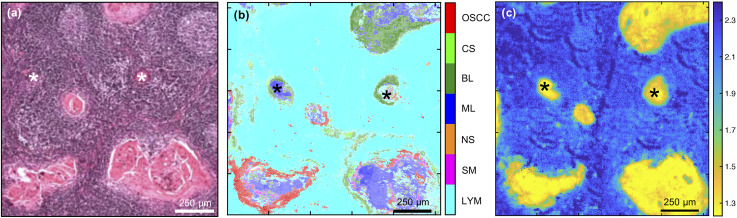
Comparison of (a) H&E image with (b) FTIR-HS multi-class combination pseudo-colour image of metastatic oral cancer and (c) FTIR ratio at 1252 cm^−1^/1285 cm^−1^. Asterisks indicate small tumour-cell aggregates.

Detection of lymph node metastases was also attainable with the multi-class MLA (Fig. 4) and both this and the single metric 1252 cm^−1^/1285 cm^−1^ were able to identify variably sized, tumour-cell aggregates. The multi-class MLA analysis suggests that the core and periphery of the metastatic deposits correspond to the maturation layers of the oral epithelium and primary OSCC or progenitor layers of the oral epithelium, respectively [compare Fig. 4(b) with Fig. 3(b) and (d)].

## Discussion

The application of the MLA to FTIR spectral images of tissue has previously identified a single metric, 1252 cm^−1^/1285 cm^−1^, that was able to identify metastatic OSCC in lymph nodes with very high sensitivity and specificity.^14^ The multi-class analysis applied in the present investigation enabled discrimination of three epithelial (OSCC, BL, ML) and four non-epithelial tissues with sensitivities between 82% and 96% and specificities between 83% and 99%. The most important wavenumbers involved in the top five ranked discriminating metrics for each tissue type were, however, tightly grouped and, in each case, a small number of wavelengths were combined in several ways to form different metrics. This suggests that the MLA is focussing on small changes in the spectral profiles of different tissue types. This is important since there is disagreement in the literature on FTIR analysis as to the wavenumbers that are characteristic of OSCC.^7–10,19–21^ Further clarification could result from analysis of a larger cohort.

The 1252 cm^−1^/1285 cm^−1^ metric was able to identify OSCC in the context of invasive primary tumour, though the multi-class analysis was better at discriminating OSCC from oral epithelium because the 1252 cm^−1^/1285 cm^−1^ metric also highlighted the maturation layers in the latter. Subtle differences could be seen between the identification of the centres (cores) of tumour-cell aggregates or maturation layers of the oral epithelium (confidently identified by the metric – bright yellow colour in Fig. 1) and periphery (front) of tumour-cell aggregates or progenitor layers of the oral epithelium (less confidently identified by the metric – green colour in Fig. 1). By comparison, non-epithelial tissues were mostly identified (blue) except for blood vessels (green). Thus, it is likely that the metric identified epithelial cells in general rather than cancer *per se*.

Discrimination between tissue types by the multi-class MLA was relatively robust, but the training sample set was small and caution should be applied in extrapolating this result to OSCC in general. For instance, while the progenitor (basal/parabasal) layers in the oral epithelium could be identified and distinguished from the maturation (spinous/keratinised) layers in the same tissue, the outline of the former defined by the MLA differed somewhat between the H&E and the FTIR-HS images. Similarly, the FTIR-HS pseudo-colour image of the pre-existing stroma underneath the progenitor layers suggests a more pronounced inflammatory reaction than in the adjacent H&E image, where it appeared patchy and of a low density. Although serial sectioning had been applied, the differences may have been influenced by the thickness of the sections, which precludes an exact correspondence. Obviously, the technique requires additional refinement before clinical utility can be considered.

An interesting observation of the multi-class MLA analysis was that in contrast with the single FTIR-HS pseudo-colour images of primary OSCC, the lymph node metastases showed a more complex pseudo-colouring combining features of OSCC, BL and ML. Analysis of a larger cohort of cases would be necessary before drawing biological inferences. It is, however, observed that these intriguing differences were not observed with the 1252 cm^−1^/1285 cm^−1^ metric and the latter metric is adequate and possibly preferable in individual cases.

It is tempting to speculate on the incorporation of the 1252 cm^−1^/1285 cm^−1^ metric into an instrument for use in a clinical setting as an intra-operative decision tool for sentinel lymph node biopsy in OSCC. Currently this engenders time consuming processing for routine histopathology effecting a delay. With some patients needing a second operation for completion of neck dissection, intra-operative decisions could confer a significant clinical advantage. However, benign epithelial inclusions are known to occur in cervical lymph nodes and the finding of the present investigation that the metric is a marker of epithelial cells rather than cancer *per se* supports the need for caution and a further, larger, study incorporating samples with such inclusions.

## Conclusions

The multi-class analysis was able to discriminate each of three epithelial and four non-epithelial tissue types with specificities and sensitivities in excess of 82%, but the training sample set was small and caution should be applied in extrapolating the observations to OSCC in general.

The most important wavenumbers involved in the top five ranked discriminating metrics for each tissue type were tightly grouped, with the 1562 cm^−1^/1539 cm^−1^ metric being identified as the best at discriminating OSCC from other tissue types. The technique requires additional refinement before any clinical utility can be considered.

The 1252 cm^−1^/1285 cm^−1^ metric that was successful^14^ in discriminating between lymph node tissue and metastatic OSCC was also able to discriminate primary OSCC from the stromal immune/inflammatory reaction and other, non-epithelial, cells is a small sample set. However, it is less discriminating in the context of cancer *vs.* oral epithelium, highlighting similarities between the maturation layers of the latter and cores of tumour-cell aggregates and, to a lesser degree, the progenitor layers of the oral epithelium and periphery of tumour-cell aggregates. It is likely that this specific metric identifies epithelial cells rather than cancer *per se* though it is clearly an effective identifier of OSCC lymph node metastases.

## Author contributions

BGE designed the experiment, collected the FTIR data, used the MLA, prepared the figures, analysed the data, wrote MATLAB scripts used for analysis of FTIR images and prepared the first draft. JI developed the MLA and analysed the data. CAW wrote MATLAB scripts to analyse FTIR data. SAJ developed the protocol for dewaxing samples. CIS designed the experiment, helped with experiments and instrumentation, prepared the figures, analysed the data, administrated the project and prepared the first draft. PJG prepared the specimens, sectioned and stained tissue samples for imaging. PG provided access to the FTIR Imaging microscope, supervised the FTIR experiments and mentored BGE. RJS provided the clinical methodology, supervised the work, obtained the funding and administrated the project. SDB developed the MLA, analysed the data, supervised the work, obtained the funding, administrated the project and prepared the first draft. AT designed the experiment, classified and annotated the stained samples for the supervised machine learning. JMR designed the experiment, selected the tissue samples, analysed the data, supervised the work, obtained the funding, administrated the project and prepared the first draft. PW designed the experiment, analysed the data, supervised the work, obtained the funding, administrated the project and prepared the first draft. All authors were involved in a critical review and edit of the paper.

## Data availability statement

The data will be available *via* the University of Liverpool Data Catalogue (https://doi.org/10.17638/datacat.liverpool.ac.uk/2206).

## Conflicts of interest

There are no conflicts to declare.

## Supplementary Material

AN-148-D3AN00258F-s001
