# Supplementary material for: Metric-based analysis of FTIR data to discriminate tissue types in oral cancer
Source: Analyst. 2023 Apr 10;148(9):1948–53. doi: 10.1039/d3an00258f (PMC10152457; doi:10.1039/d3an00258f)
Supplement: AN-148-D3AN00258F-s001 [file AN-148-D3AN00258F-s001.pdf]

Electronic Supplementary Material (ESI) for Analyst.
This journal is © The Royal Society of Chemistry 2023

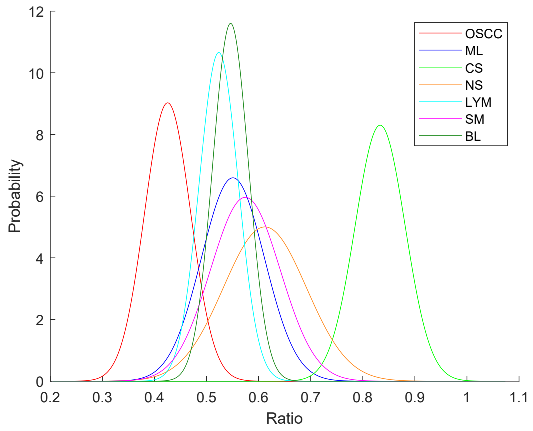

Fig. S1: Histograms showing the distribution for each tissue type at the top ranked metric for OSCC (i.e. 1562 cm^{-1}/1539 cm^{-1}; Table 2).

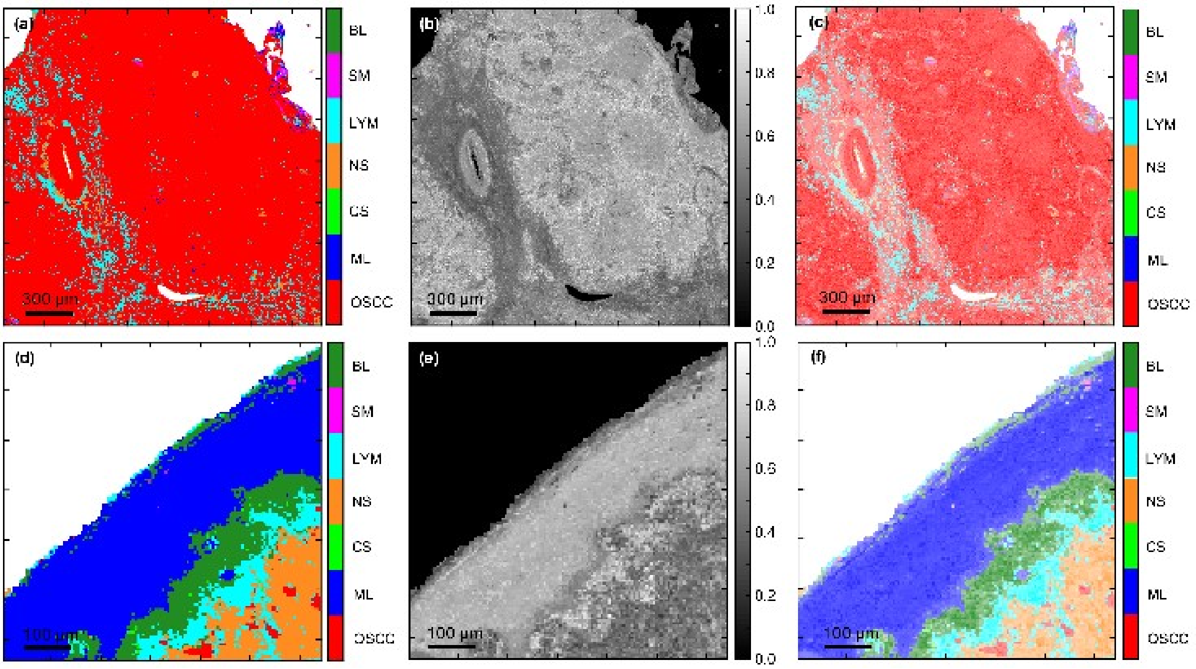

Fig. S2: Pseudo-colouring of FTIR-HS images of oral cancer (top) and oral epithelium (bottom) (a) and (d): pseudo-colour images labelled by the MLA; (b) and (e): confidence level of the MLA in labelling each pixel and (c) and (f) a combination image with the saturation of each pixel corresponding to the confidence of the MLA in identifying the tissue type. OSCC: oral squamous cell carcinoma; CS: tumour stroma with immune/inflammatory reaction; BL: progenitor layers of oral epithelium; ML: maturation layers of oral epithelium; NS: pre-existing stroma; SM: submucosal components; LYM: lymphoid node tissue.
